# Imaging Case: NK/T-Cell Lymphoma, Nasal Type

**Published:** 2015-11

**Authors:** Srini Vasan, Prathiba Rajalakshmi, Sanjay Thulkar

**Affiliations:** 1 *Department of Radio-diagnosis, All India Institute of Medical Sciences, New Delhi, India. Tel: 91-9677146026, E-mail: drksvasan@gmail.com*

**Fig 1:**
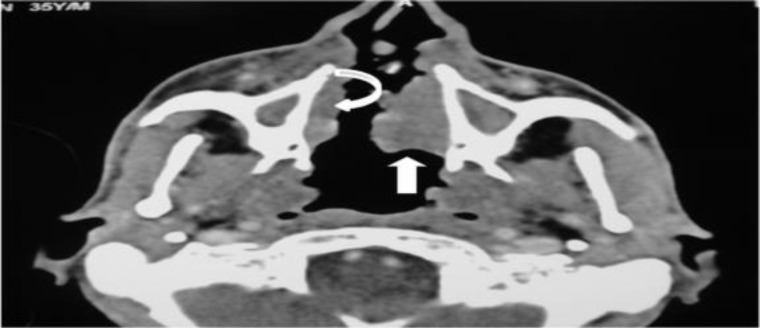
Axial image at the level of nasal cavity shows an irregular soft tissue mass in the left (arrow) and right (curved arrow) lateral walls of nasal cavity with complete destruction of the nasal septum

**Fig2:**
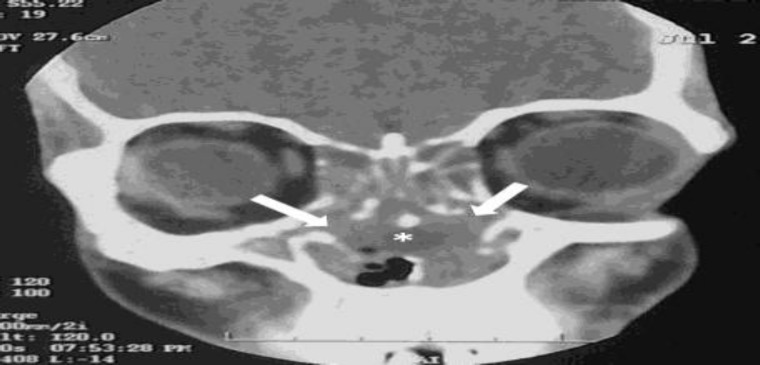
Coronal CT image shows destruction of nasal septum (asterisk) with mass filling the nasal cavity (arrows

Peripheral T-cell lymphomas are a group of heterogeneous disorders and according to WHO classification, are categorized into nodal and extranodal forms. NK/T-cell lymphoma, nasal type, is a subtype of extranodal peripheral T-cell lymphoma and commonly presents as a midfacial destructive lesion. This disorder is more prevalent in Asia and South America and has a strong association with Epstein Barr Virus infection. Invasion of vessel walls by lymphoid cells, which is known as angiocentricity, is characteristic of nasal type NK/T-cell lymphoma. The tumor cells express CD2 and CD56 antigens; but not CD3. The nasal cavity is the mostly frequently affected site. Other commonly affected sites include palate and upper airways. On cross sectional imaging, the nasal involvement is seen as a diffuse sheet-like mucosal thickening along the nasal turbinates and septum or as a destructive midline mass (Figs.1,2).

**Fig 3:**
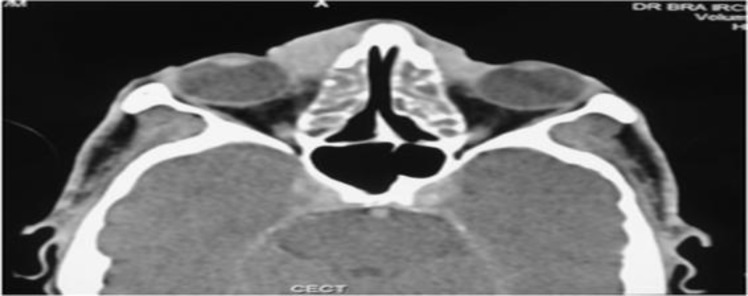
Axial image at the level of optic nerves shows a sheet like soft tissue mass at the root of nose and bilateral medial canthi with retained secretions in bilateral ethmoid sinuses

The latter form was previously described as a lethal midline granuloma or polymorphic reticulosis. The mass frequently extends into subcutaneous tissues of nasal ala and buccinator space (Fig.3).

**Fig4:**
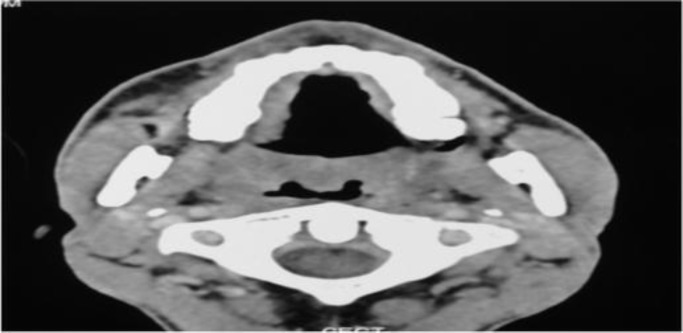
Axial section at the level of soft palate shows thickening of the soft palate and bilateral tonsils

Regional lymphadenopathy is usually not seen. The radiological differential diagnoses for a midline nasal cavity mass include squamous cell carcinoma, minor salivary gland tumor, Wegener’s granulomatosis, and fungal infections. The imaging appearances of NK/T-cell lymphoma are often indistinguishable from the above mentioned conditions. However, predilection to involve both sides of the nasal cavity and tendency to spread as a diffuse thin sheet-like soft tissue along the walls of the nasal cavity enveloping the nasal turbinates and nasal septum favour the diagnosis of NK/T-cell lymphoma. Contiguous extension into the nasopharynx, palate, upper airways, and subcutaneous tissues can also suggest the possibility of NK/T-cell lymphoma, nasal type (Fig.4).

 T-cell lymphoma, compared to B-cell lymphoma, has an aggressive course and poor prognosis. The median survival was reported to be 12 months, even in patients showing a localised disease. Extranodal NK/T-cell lymphoma is sensitive to both chemo- and radiotherapy. Methotrexate and anthracycline based chemotherapy regimen (SMILE protocol) with infield radiotherapy is the recommended protocol for treatment of NK/T-cell lymphoma.

